# Immunogenicity Characterization of Two Ancient Wheat α-Gliadin Peptides Related to Coeliac Disease

**DOI:** 10.3390/nu1020276

**Published:** 2009-12-17

**Authors:** Armando Gregorini, Mariastella Colomba, H. Julia Ellis, Paul J. Ciclitira

**Affiliations:** 1 DiPsiTer, University of Urbino “Carlo Bo”, via Ubaldini 17, 61029, Urbino, PU, Italy; 2 DiSUAN, University of Urbino “Carlo Bo”, via Maggetti 22, 61029, Urbino, PU, Italy; Email: mariastella.colomba@uniurb.it; 3 King’s College London, Division of Nutritional Sciences, Rayne Institute, St. Thomas’ Hospital, London SE1 7EH, UK; Email: julia.ellis@kcl.ac.uk (H.J.E.); paul.ciclitira@kcl.ac.uk (P.J.C.)

**Keywords:** coeliac disease, ancient wheats, α-gliadin, toxic peptides, monoclonal antibodies, *Triticum turgidum durum*

## Abstract

The immunogenic potential of α-gliadin protein from two ancient wheats was studied with reference to coeliac disease. To this aim we investigated Graziella Ra® and Kamut® (the latter is considered an ancient relative of modern durum wheat) in comparison to four durum wheat accessions (Senatore Cappelli, Flaminio, Grazia and Svevo). ELISA and Western Blot analyses - carried out by two monoclonal antibodies raised against the α-gliadin peptides p31-49 (LGQQQPFPQQPYPQPQPF) and p56-75 (LQLQPFPQPQLPYPQPQLPY) containing a core region (underlined) reported to be toxic for coeliac patients - always showed an antibody-antigen positive reaction. For all accessions, an α-gliadin gene has also been cloned and sequenced. Deduced amino acid sequences constantly showed the toxic motifs. In conclusion, we strongly recommend that coeliac patients should avoid consuming Graziella Ra® or Kamut®. In fact their α-gliadin not only is as toxic as one of the other wheat accessions, but also occurs in greater amount, which is in line with the higher level of proteins in ancient wheats when compared to modern varieties.

## 1. Introduction

Wheat is an important staple food because of its characteristics such as the high nutritional value, technical properties and the long life of the kernels. Commonly used varieties belong to tetraploid (*Triticum turgidum durum*, pasta wheat) or hexaploid (*Triticum aestivum*, bread wheat) species originating from natural hybridizations between diploid ancestors thousands of years ago. Wheat endosperm contains 8–15% of proteins, of which 80% is gluten whose components are gliadins and glutenins, each one having peptides able to induce coeliac disease (CD), an intestinal chronic disorder caused by an intolerance to gluten proteins, mainly resulting in small-intestinal mucosal injuries and nutrient malabsorption in susceptible individuals [1]. The only effective treatment available for CD patients is a strict exclusion of gluten from their diet. The detrimental consequences of gluten and/or analogous proteins (present in rye, barley and oats) consumption are well documented, showing that noncompliance to a gluten-free diet is associated with increased risk of anemia, infertility, osteoporosis and intestinal lymphoma [2]. In recent years it has become clear that CD is far more common than previously thought. Several serological screening studies from Europe, South America, Australasia and the USA have shown that approximately 0.5–1% of these populations suffer from CD. Nevertheless, most affected individuals remain undiagnosed due to an increasingly broad spectrum of clinical presentations [3]. Moreover, CD is a multifactorial disorder including both genetic and environmental factors whose relative weight is not yet fully understood. Differences in concordance rates in monozygotic (86%) and dizygotic (20%) twins strongly suggest a relevant influence of genetic factors, of which HLA (Human Leukocyte Antigen) is estimated to contribute for 40-50% to disease development [4,5]. In particular, while roughly 95% of CD patients carry HLA-DQ2 (DQA1*0501/DQB1*0201), most individuals that are not HLA-DQ2 positive express HLA-DQ8 (DQA1*0301/DQB1*0302). Both HLA-DQ2 and HLA-DQ8 have very characteristic peptide binding motifs characterized by a preference for hydrophobic and negatively charged amino acids at specific positions in peptides resulting mostly from gliadins digestion [6,7], although the coeliac toxicity of glutenins becoming increasingly appreciated [8].

According to their mobility in lactic acid PAGE (A-PAGE), gliadins can be subdivided into four subfractions: α/β-gliadins, γ-gliadins and ω-gliadins, whereas the glutenins consist of low and high molecular weight (LMW and HMW) glutenins, the latter being particularly important for the baking quality of dough. Gliadins have several unique features that contribute to their immunogenic properties. They are extremely rich in proline (P) and glutamine (Q) and, consequently, highly resistant to proteolytic degradation within the gastrointestinal tract, since gastric and pancreatic enzymes lack post-proline cleaving activities [9]. Additionally, the high glutamine content makes gliadins a good substrate for tissue transglutaminase (tTG), an enzyme constitutively expressed in the *lamina propria* playing a role in tissue repair. Under physiological conditions, tTG can also convert (during the deamidation process) glutamine into the negatively charged glutamic acid (E), leading to enhanced immunogenicity of the resulting modified peptides, which can preferentially bind to HLA-DQ2 or HLA-DQ8 [10,11].

Deamidation is most likely a crucial event in the generation of a full-blown gluten-specific T cell response and concomitant CD development. Many gluten peptides with T cell stimulatory capacity have been identified in the α/β-gliadins, γ-gliadins and low and high molecular weight glutenins [12,13].

Recent work has shown that in addition to a gluten specific T cell activation, there is also activity of the innate immune system, mediated by interleukin 15 (IL15) [14] which may be invoked by gliadin peptides, particularly α-gliadin 31-49 that do not stimulate small intestinal T cells [15] but which cause *in vitro* [16,17] and *in vivo* coeliac toxicity [10]. *In vivo* instillation of HMW glutenins caused an early release of IL15 in coeliac patients [8].

Attempts to generate wheat (and other cereals) with absent or reduced immunogenicity by selective breeding or genetic modifications to detoxify gluten by the introduction of amino acid substitutions are still in progress. Currently, available wheat varieties are the result of field selections based on several criteria including: (i) high yield (based on a system of high inputs, i.e artificial fertilizers); (ii) disease resistance and (iii) technological qualities, e.g., bread- or pasta-making qualities; while there is little emphasis on taste and nutrition. In the last decades, ancient populations of local varieties have been considerably reduced as a result of the “green revolution” and the diffusion of the new varieties of wheat. However, some ancient wheats (which have not been subjected to major genetic improvement) have recently been re-introduced to prevent the loss of often locally grown grain varieties, maintain biodiversity and avoid food allergies or intolerances. In fact it has been suggested that certain varieties of ancient wheats would appear to have fewer toxic motifs and therefore might be better suited to be introduced into the diets of people who suffer from food intolerances or allergies [18,19].

In this regard, the CD-immunogenic properties of gliadins from the ancient wheats Graziella Ra®, which appeared on the market a few years ago and was uncharacterized from this point of view, and Kamut®, which is considered an ancient relative of durum wheat, have been investigated. To this aim, a comparative analysis including one traditional wheat (Senatore Cappelli) and three modern accessions (Flaminio, Grazia and Svevo) was carried out. In particular, we investigated the α-gliadin peptides p31-49 (LGQQQPFPQQPYPQPQPF) and p56-75 (LQLQPFPQPQLPYPQPQLPY) - a T cell stimulatory epitope whose core region (underlined) is toxic for coeliac patients as a result of tTG deamidation (PQLPY→PELPY) - by ELISA and Western Blot using two specific monoclonal antibodies (mAbs). Moreover, for all the accessions, α-gliadin genes, once cloned and sequenced, were analysed to assess their variability and search for toxic motifs into the corresponding deduced amino acid sequences.

## 2. Results and Discussion

In order to investigate whether α-gliadins from the ancient wheats Graziella Ra® and Kamut® would contain the two main toxic peptides (p31-49: LGQQQPFPQQPYPQPQPF; and p56-75: LQLQPFPQPQLPYPQPQLPY) related to CD, we compared them to a traditional strain (Senatore Cappelli) and to three modern varieties (Flaminio, Grazia and Svevo), usually employed in pasta- or bread-making, by two complementary approaches: α-gliadin peptides analysis performed by standard proteomic techniques (ELISA and Immunoblotting); and molecular analysis based on α-gliadin gene sequencing.

### ELISA

**Figure 1:**
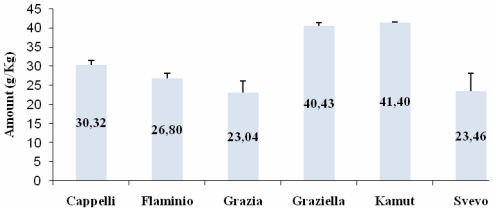
Total gliadin determination by a two step sandwich ELISA. All values are reported as mean ± SD from three independent experiments.

In a preliminary step we assessed the amount of total gliadin in all the accessions using a commercially available gluten test kit (Gliadin ELISA kit, Immunotech, Czech Republic). For each sample, total gliadin content (mg/Kg) was calculated accordingly to the formula reported in manufacturer’s instructions (for details see the Experimental section). As shown in Figure 1, Kamut® (41.40 g/Kg) and Graziella Ra® (40.43 g/Kg) kernels had the greater amounts of gliadin, followed by Senatore Cappelli (30.32 g/Kg), Flaminio (26.80 g/Kg), Svevo (23.46 g/Kg) and Grazia (23.04 g/Kg).

**Figure 2:**
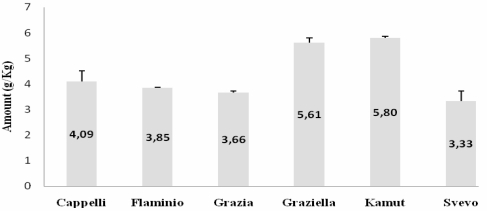
A-gliadin peptidep31-49 (LGQQQPFPPQQPYPQPQPF) quantitative determination by PN3 mAb. All values are reported as mean ± SD from three independent experiments.

**Figure 3:**
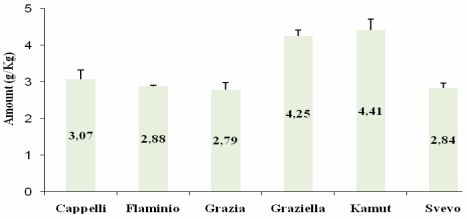
A-gliadin peptide p56-75 (LQLQPFPQPQLPYPQPQLPY) quantitative determination by CDC-5 mAb. All values are reported as mean ± SD from three independent experiments.

Subsequently, we tested each gliadin extract against the PN3 and CDC-5 mAbs, using gliadin from Sigma-Aldrich (UK) as a standard for quantitative analysis. As illustrated in Figure 2 and Figure 3, our experiments showed an antigen-antibody positive reaction in every single case. Analyses gave similar outcomes for both mAbs. Particularly, two subsets could be evidenced, the first of which grouping Graziella Ra® and Kamut®, quite rich in toxic peptides, and the second consisting of the remaining relatively poorer accessions. Noteworthily, Senatore Cappelli confirmed to give an antibody positive reaction stronger than the three modern cultivars under study. 

### Western Blot

**Figure 4:**
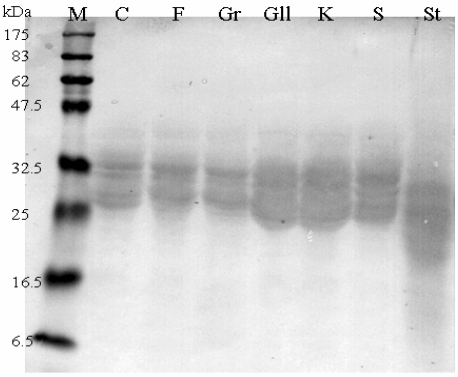
Immunoblotting of α-gliadin extracts with PN3 mAb. M: Prestained Protein Marker (New England Biolabs); C: Senatore Cappelli; F: Flaminio; Gr: Grazia; Gll: Graziella Ra®; K: Kamut®; S: Svevo; St: Standard Gliadin (Sigma-Aldrich).

SDS-PAGE and Western Blot of α-gliadin from each accession with PN3 and CDC-5 (data not shown) mAbs gave similar patterns showing thicker signals in correspondence of the two ancient wheats, confirming ELISA results (Figure 4).

### Α-Gliadin Gene Sequences Description and Variability

Α-gliadin genes from all the accessions were cloned and sequenced. On the whole for all the accessions under study we characterised several alleles, broadly grouped into two subsets, identified as short (S) or long (L), respectively. S alleles ranged from 828 bp to 867 bp, while L alleles were between 894 bp and 963 bp. Every accession showed at least one allele of each type. Moreover, several α-gliadin alleles containing one or more internal stop codons were observed. In line with previous studies, we referred to them as pseudogenes, although we cannot predict from the genomic data whether a subset is being expressed [20]. All sequences have been submitted to GenBank database (GQ999806-GQ999831) (Table 1).

**Table 1:** Number of obtained unique full Open Reading Frame (ORF) and sequences with one or more stop codons (pseudogenes) from wheats under study. GenBank accession numbers are reported between brackets.

Wheat accessions	Full-ORF	Pseudogenes	Total
Senatore Cappelli	2 (GQ999806-GQ999807)	3 (GQ999818-GQ999820)	5
Flaminio	2 (GQ999808-GQ999809)	1 (GQ999821)	4
Grazia	2 (GQ999810-GQ999811)	2 (GQ999822-GQ999823)	4
Graziella	2 (GQ999812-GQ999813)	2 (GQ999824-GQ999825)	4
Kamut	2 (GQ999814-GQ999815)	3 (GQ999829-GQ999831)	6
Svevo	2 (GQ999816-GQ999817)	3 (GQ999826-GQ999828)	5
Total	12	14	26

The α-gliadin L genes alignment showed five different haplotypes (Flaminio_L and Kamut_L clustered together), defined by 79 polymorphic sites (S = 79) including 80 substitutions (Eta = 80, 56 transitions and 24 transversions) and 69 insertions/deletions. Parsimony informative sites = 11. Haplotype diversity (Hd) was 0.933 ± 0.122. Nucleotide diversity (π) was 0.032 ± 0.011 and 0.033 when corrected according to Jukes and Cantor (π_JC_). The average number of nucleotide differences (k) was 28.800. Assessed sequence similarity was 89%. Tajima’s D was −1.148, p > 0.10 (not significant), hence the null hypothesis of neutral evolution of these DNA sequences was not rejected. Tajima's D is a statistical test created to distinguish between a DNA sequence evolving randomly and one evolving under a non-random process (under selection). A randomly evolving DNA sequence contains mutations with no effect on the fitness and survival of an organism. The randomly evolving mutations are called "neutral", while mutations under selection are "non-neutral". Tajima’s test of neutrality is based on the fact that under the neutral model estimates of the number of segregating/polymorphic sites and of the average number of nucleotide differences are correlated. If the value of D is too large or too small, the neutral 'null' hypothesis is rejected.

The α-gliadin S genes alignment showed six different haplotypes, defined by 149 polymorphic sites (S = 149) including 149 substitutions (Eta = 149, 98 transitions and 51 transversions) and 63 insertions/deletions. Parsimony informative sites = 48. Haplotypes revealed to be restricted to unique accessions. Haplotype diversity (Hd) was 1.000 ± 0.096. Nucleotide diversity (π) was 0.074 ± 0.012 and 0.079 when corrected according to Jukes and Cantor (π_JC_). The average number of nucleotide differences (k) was 60.600. Assessed sequence similarity was about 83%. Tajima’s D was −0.462, p > 0.10 (not significant), hence the null hypothesis of neutral evolution of these DNA sequences was not rejected.

**Figure 5:**
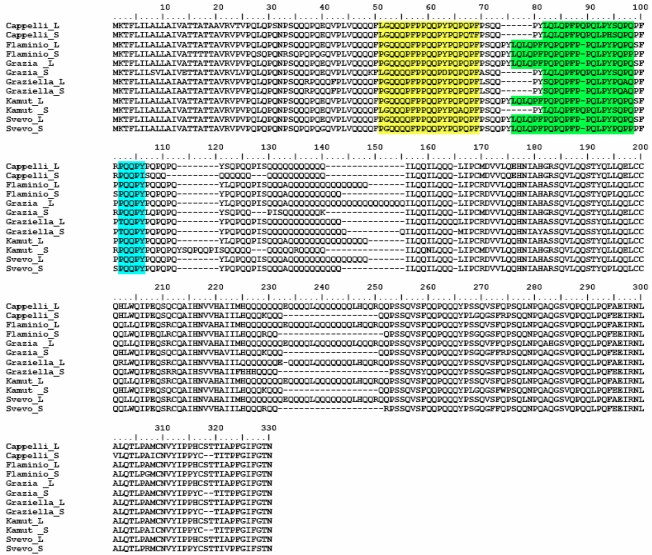
Alignment of deduced L and S α-gliadin isoforms of each wheat accession. Epitopes recognized by PN3 (yellow highlighted) and CDC-5 (green highlighted) mAbs are shown. An additionalPQQPY motif (blue highlighted) is indicated.

Finally, each allele was translated (with a BioEdit dedicated option) into the corresponding protein. Proteins alignment was analysed searching for the two epitope motifs which were found in all deduced amino acid sequences. Moreover, for all examined isoforms, another PQQPY string, whose immunogenic significance should be further investigated, was detected (Figure 5).

**Figure 6:**
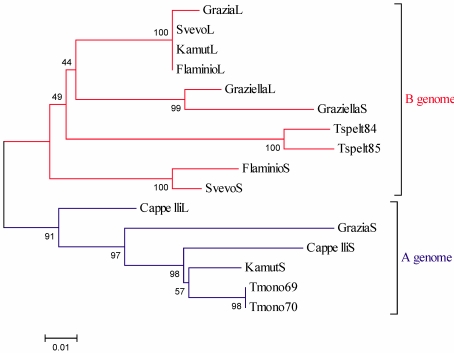
Neighbor-Joining tree of α-gliadin deduced amino acid sequences. The optimal tree with the sum of branch length = 0.533 is shown. The percentage of replicate trees in which the associated taxa clustered together in the bootstrap test (1,000 replicates) are shown next to the branches. The evolutionary distances were computed using the Poisson correction method and are in the units of the number of amino acid substitutions per site. All positions containing gaps and missing data were eliminated from the dataset (Complete deletion option). L and S refer to long and short deduced amino acid sequences; Tspelt: *Triticum speltoides*; Tmono: *Triticum monococcum*.

A phylogenetic analysis of the translated full-ORF (Open Reading Frame) α-gliadin genes demonstrated a clustering of the sequences according to their genome of origin. Indeed, by comparison to diploid ancestors, *T.* *monococcum* (A genome) and *T. speltoides* (closely related to B genome), the analysed sequences formed two separate clusters (subtrees) of relatively closely related isoforms in the phylogenetic tree (Figure 6).

In the present study we characterized α-gliadin from Graziella Ra® and Kamut®, in order to investigate the controversial hypothesis suggesting ancient grains might show lower immunogenic properties and therefore the possibility to introduce them in the diet of wheat-sensitive people, including coeliac patients.

By using a commercially available gluten test kit, we determined that Graziella Ra® and Kamut® have relatively greater amount of α-gliadin when compared with the other accessions under study. Taking into account that gluten represents about 80% of the entire protein reservoir in wheat and that it is composed by gliadins and glutenins, present results would validate the producers’ claims that ancient wheats are endowed with kernels usually bigger and richer in proteins than common wheats. Interestingly, Senatore Cappelli showed an amount of α-gliadin that is in-between ancient and modern accessions, which supports the placing it as a traditional (*i.e.*, the first selected) wheat.

When employing a two step indirect ELISA with PN3 and CDC-5, specifically raised against p31-49 and p56-75, similar patterns were observed for both mAbs. Our data demonstrate the occurrence of the two peptides in all the accessions analysed. In particular, Graziella Ra® and Kamut® showed the highest values, in contrast with the “low immunogenicity” hypothesis. Such findings were also confirmed by immunoblotting, as revealed by marked hybridization signals located on Graziella Ra® and Kamut® lanes in developed films (see Figure 4).

As far as molecular analysis is concerned, the occurrence of two alleles of different sizes (L and S type) for each accession, is in line with the relatively high number of α-gliadin alleles reported for diploid wheat ancestors (*T. speltoides* and *T. monococcum*) and hexaploid (*T. aestivum*) species [21]. Observed L and S alleles differ from each other, at least in this case, mostly in the length of few CAA-rich regions. The amplification of α-gliadin alleles from A or B genome employing the same primers pair can be considered as a result of the typical structure of the gene. Indeed, the fact that the sequences at the 5' end (signal peptide) and 3' end of the genes are highly conserved enables to obtain different members of the gene family when using a PCR-based approach. Moreover, we encountered also a consistent percentage of pseudogenes all showing the C→T substitution as the most common single-base change, contributing to the generation of stop codons. Such findings agree with previous observations that at least 50% of the α-gliadin genes are pseudogenes [21,22]. Interestingly, Tajima’s test results seem to suggest that analysed α-gliadin genes mainly undergone neutral evolution. To better investigate the nature of evolutive forces, we computed the number of synonymous (K_s_) and non-synonymous (K_a_) substitutions per site for all full ORF and pseudogene obtained sequences. A synonymous (or silent) substitution is the substitution of one base for another in an exon of a gene, such that the amino acid sequence produced is not modified. Otherwise, a non synonymous substitution results in an altered sequence in a polypeptide or functional RNA. In the study of evolution, DNA sequences are more informative than protein sequences since a large part of DNA is not translated and there is degeneracy of the genetic code. Furthermore, according to King and Jukes [23,24] because of degeneracy of the genetic code, a certain proportion of nucleotide substitutions in protein-coding genes are expected to be silent and result in no amino acid substitutions; and, since synonymous substitutions should be neutral, K_s_ rate should be higher than K_a_ rate if neutral theory is correct. Noteworthy, the mean K_a_/K_s_ ratio for the full ORFs was lower than that of the pseudogenes (0.368 ± 0.006 *vs* 0.417 ± 0.116), thus demonstrating the occurrence of a statistically significant (t test, p < 0.05) excess of synonymous substitutions in genes when compared to pseudogenes. These findings are in line with the neutral evolution hypothesis as also suggested by Tajima’s test results.

When analysing the deduced amino acid sequences we found a high degree of similarity (about 75% and 86% for S and L deduced isoforms, respectively). Moreover, the p31-49 and p56-75 epitopes occurred in all proteins and, although slightly modified (due to a few amino acid substitutions) with respect to available GenBank sequences (ABB92632; ABB92633; CAI35909), they constantly showed highly conserved toxic core regions (PQQPY; PQLPY). The antibody PN3 has been shown to bind in the region QQQPFP of the peptide p31-49. This peptide does not have small intestinal T cell stimulatory properties, but has been shown active to the innate immune system in coeliac patients [17] and to be toxic *in vivo* [10]. The exact sequence within the T cell stimulating peptide p56-75 identified by the antibody CDC-5 is not known at present. However, although further studies to analyse T cell clone reactivity would be of importance, the association of proteomic (ELISA and Immunoblotting) and genomic (molecular analysis) investigations, demonstrated the potential high immunogenicity and toxicity to CD patients of all the accessions under study, including the two ancient wheats. 

## 3. Experimental Section

### Wheat Accessions

Graziella Ra® is an ancient durum wheat (*Triticum turgidum durum*), organically grown only in the Marche region by the Alce Nero Cooperative (Isola del Piano, PU, Marche region, Italy), that appeared on the market a few years ago due to its good taste and fine pasta-making quality. It derived from kernels taken from an Egyptian tomb and was brought to Italy in the late 70’s (Graziella Ra® history is fully reported at http://www.alcenerocooperativa.it). Up to now, Graziella Ra® genome has never been characterized; hence, the analysis of the CD-immunogenic properties of its α-gliadin appeared to be a very intriguing issue. 

Kamut® is an ancient type of wheat related to the durum subspecies. At present its systematics is still uncertain. In fact, originally, it was classified as *Triticum turgidum polonicum* (Polish wheat), but also suggested by some to be *Triticum turgidum turanicum* (oriental or Khurasan wheat), or being in fact *Triticum turgidum durum* (durum wheat). Due to its ancient origin and easy availability, Kamut® was chosen to be compared to Graziella Ra®. For the present study, a small amount (100 g) was kindly provided by “Molini del Conero by Agriconero Srl” (Osimo, AN, Italy).

Grazia, Flaminio, Svevo (modern varieties) and Senatore Cappelli (a traditional strain, namely the first “chosen” variety selected by N. Strampelli) are all *Triticum turgidum durum* and were provided by “Alce Nero Cooperative” (Isola del Piano, PU, Italy).

### Gliadin Extraction

For each accession, wheat kernels were ground in an electric grinder to produce a homogenous sample powder. Subsequently, gliadins were extracted following standard protocols: briefly, 1 g of powder was transferred to 10 mL of 60% (v/v) ethanol for 30 min, shaking vigorously. After centrifugation (3,000 g x 15 min) at room temperature (RT), the supernatant was recovered and stored frozen (−20 °C). As a standard, whole gliadin from Sigma-Aldrich (G3375) was chosen because it compares well to the European gliadin standard [25].

### Monoclonal Antibodies

To determine α-gliadin amount in extracts from all accessions and in Sigma gliadin, two monoclonal antibodies (mAbs), PN3 and CDC-5, were used. The PN3 mAb [26] was raised against a synthetic peptide equivalent to the amino acids 31–49 (LGQQQPFPPQQPYPQPQPF) of α-gliadin, which has been shown to cause mucosal damage to the small bowel of coeliac patients, both *in vivo* and *in vitro* [10,16,17]. This mAb was used at 1:800 and 1:1,000 dilutions in Western Blot and Indirect ELISA, respectively.

CDC-5 mAb [27] was raised against a synthetic peptide equivalent to the amino acids 56-75 (LQLQPFPQPQLPYPQPQLPY) of α-gliadin, which has been indicated as highly immunogenic in adult coeliac patients. CDC-5 was used at 1:1,500 and 1:2,000 dilutions in Western Blot and Indirect ELISA, respectively.

### Enzyme-Linked ImmunoSorbent Assay (ELISA)

For each accession, total gliadin was assessed by a commercial two immunological step sandwich assay type, the Gliadin ELISA kit (IM3717, Immunotech, Czech Republic) accordingly to the manufacturer’s instructions. 

Subsequently, α-gliadin was determined by indirect two-steps ELISA following standard procedures [28]. Briefly, 96-microwells plates were coated with samples (diluted 1:3,000) and standards (Sigma gliadin at concentrations ranging from 0 ng/mL to 1000 ng/mL) overnight at 4 °C in the dark, incubated with murine monoclonal antibodies (PN3 or CDC-5) for one hour and goat anti-mouse immunoglobulin (IgG, H + L) conjugated to horseradish peroxidase (Pierce Biotechnology, USA) diluted 1:5,000, for one hour. The substrate, tetramethylbenzidine (TMB), was added to plates and left 15 minutes at RT in the dark. Finally the absorbance at 450 nm was determined using an ELISA plate reader (SunriseII, Tecan, Italy).

Results were read off a semi-logarithmic calibration curve, constructed as the dependence of measured absorbance values (vertical axis - linear scale) of corresponding calibrators (standard Sigma-gliadin), range 0–1,000 ng/mL (horizontal axis – logarithmic scale). For each extract, concentrations were recalculated to the content of gliadin expressed in mg/Kg of dry substance according to the formula:

**C[mg/Kg]=[*C_kk_ (ng/mL) x extract_volume (mL) x* dilution_factor] / [1,000 x dry_substance_rate x weight (g)]**

where C_kk_, concentration reading at 450 nm; dry_substance_rate = 0.9 (*i.e.*, 90% of dry substance in sample). 

### SDS-PAGE and Western Blot

Whole gliadins (10 mg/mL each) from experimental wheats and Sigma gliadin (1 mg/mL) were separated by SDS-PAGE on a 12.5% acrylamide gel in reducing condition [29]. Afterwards, proteins were transferred on a 0.45 μm nitrocellulose membrane (Whatman, Germany), incubated with CDC-5 or PN3 monoclonal antibodies, diluted 1:800 and 1:400 respectively, and visualized with goat anti-mouse immunoglobulin (IgG, H + L) labelled with horseradish peroxidase (Pierce Biotechnology, USA) diluted 1:20,000, using SuperSignal West Pico Chemiluminescence Substrate (Pierce Biotechnology, USA). 

### DNA Extraction, Amplification, Cloning and Sequencing

Fifteen seeds of each cultivar germinated in the dark for two days. The seedlings were grown in daylight for seven days. The leaf tissues - sampled at the four-leaf stage from ten different plants per accession – were immediately frozen in liquid nitrogen and ground in a mortar with a pestle. 50 mg of powder was used for DNA extraction following the cetyltrimethylammonium bromide (CTAB) buffer protocol with slight modifications [30]. DNA quality was tested by 0.8% agarose gel electrophoresis.

Primers to amplify α-gliadin genes from genomic DNA using PCR, were designed on the conserved sequences at the 5’ and 3’ end of the coding region of the α-gliadin gene sequences obtained from the GenBank database (DQ296195, DQ296196 and AJ870965):

forward primer: 5’- ATG AAG ACC TTT CTC ATC C – 3’
reverse primer: 5’- YYA GTT RGT ACC GAA GAT GCC – 3’

PCR amplification was carried out using an automated DNA thermal cycler (ThermoHybaid, Celbio, Italy) and a high fidelity Pfu DNA Polymerase (Promega, Madison, WI, USA) as follows: 95 °C for 2 min; 95 °C for 1 min, 60 °C for 30 sec, 72 °C for 2 min (30 cycles); 72 °C for 5 min. The reaction product was visualized by electrophoresis on a 1.2% agarose gel containing tris-borate-EDTA buffer and ethidium bromide (0.5 μg/mL). An aliquot (1 μL) of the PCR product was inserted into a pCR 4-TOPO vector by the TA-cloning system and transformation was performed on *Escherichia coli* TOP10 cells following the manufacturer’s instructions (Invitrogen, Carlsbad, CA, USA). The selected transformants were analysed for presence and correct orientation of the insert by PCR, grown in LB medium overnight and purified by the Wizard Plus SV minipreps kit (Promega). Finally, sequencing of plasmid inserts was done by using automated DNA sequencers at Eurofins MWG Operon (Germany).

Sequences were visualized with BioEdit Sequence Alignment Editor version 7.0.5.3 [31], aligned with the ClustalW option included in this software and double checked by eye. Standard measures of nucleotide polymorphism [haplotype diversity (Hd), mean pairwise differences (k), nucleotide diversity (π)] and Tajima’s test of neutrality [32] were computed using DNAsp version 5 [33]. Deduced amino acid sequences were obtained and analysed by BioEdit dedicated options. Phylogenetic analysis of α-gliadin deduced amino acid sequences alignment were performed using MEGA version 4.0.2 [34]. The evolutionary distances were computed using the Poisson correction method [35] and are in the units of the number of amino acid substitutions per site. All positions containing gaps and missing data were eliminated from the dataset (Complete deletion option). Phylogenetic trees were reconstructed using the Neighbour-Joining procedure [36]. Support for the internodes was assessed by bootstrap percentages [37] after 1,000 resampling steps. Α-gliadin deduced amino acid sequences were compared to deduced amino acid sequences of homologous genes from diploid species that represent the ancestral genomes of durum wheat, *T. monococcum* (A genome) and *T. speltoides* (considered as very close to the ancestor of B genome) available in GenBank database (DQ002569-DQ002570; DQ002584-DQ002585). Statistical analysis was performed by a two tailed t test for independent data.

## 4. Conclusions

In this study we aimed to test whether α-gliadin from two ancient wheats could be considered less “toxic” for coeliac patients. Our results demonstrate that (i) the ancient wheats Graziella Ra® and Kamut® have greater amounts of α-gliadin than modern accessions; (ii) α-gliadins from such ancient wheats show a strong and specific binding reaction to anti-p31-49 and anti-p56-75 mAbs, thus suggesting the occurrence of epitopes with proven coeliac toxicity; (iii) α-gliadins from ancient and modern wheat accessions analysed share common features and properties both from immunological and molecular point of view. In conclusion, we suggest that Graziella Ra® and Kamut® are potentially as toxic as modern wheats with reference to CD and, therefore, although clinical trials are still necessary, we strongly recommend that they should not be introduced in the diet of coeliac patients.
